# Jordan Field Epidemiology Training Program: Critical Role in National and Regional Capacity Building

**DOI:** 10.2196/mededu.9516

**Published:** 2018-04-11

**Authors:** Mohannad Al Nsour, Ibrahim Iblan, Mohammed Rasoul Tarawneh

**Affiliations:** ^1^ Eastern Mediterranean Public Health Network Amman Jordan; ^2^ Community Medicine Jordan Ministry of Health Amman Jordan

**Keywords:** field epidemiology, training program, education, capacity building, disease outbreaks, public health surveillance, epidemiological monitoring, Jordan

## Abstract

Field Epidemiology Training Programs (FETPs) are 2-year training programs in applied epidemiology, established with the purpose of increasing a country’s capacity within the public health workforce to detect and respond to health threats and develop internal expertise in field epidemiology. The Jordan Ministry of Health, in partnership with the US Centers for Disease Control and Prevention, started the Jordan FETP (J-FETP) in 1998. Since then, it has achieved a high standard of success and has been established as a model for FETPs in the Eastern Mediterranean Region. Here we describe the J-FETP, its role in building the epidemiologic capacity of Jordan’s public health workforce, and its activities and achievements, which have grown the program to be self-sustaining within the Jordan Ministry of Health. Since its inception, the program’s residents and graduates have assisted the country to improve its surveillance systems, including revising the mortality surveillance policy, implementing the use of electronic data reporting, investigating outbreaks at national and regional levels, contributing to noncommunicable disease research and surveillance, and responding to regional emergencies and disasters. J-FETP’s structure and systems of support from the Jordan Ministry of Health and local, regional, and international partners have contributed to the success and sustainability of the J-FETP. The J-FETP has contributed significantly to improvements in surveillance systems, control of infectious diseases, outbreak investigations, and availability of reliable morbidity and mortality data in Jordan. Moreover, the program has supported public health and epidemiology in the Eastern Mediterranean Region. Best practices of the J-FETP can be applied to FETPs throughout the world.

## Introduction

The Field Epidemiology Training Program (FETP) is a 2-year training program in applied epidemiology, modeled after the US Centers for Disease Control and Prevention’s (CDC) Epidemic Intelligence Service [1]. The purpose of the FETP is to increase the epidemiologic capacity of a country’s public health workforce to detect and respond to health threats and develop internal expertise in field epidemiology [2]. FETPs’ curricula aim at improving public health systems and developing professional skills to ensure the country meets surveillance and response requirements. The programs are established within national ministries of health and may access technical assistance from the CDC.

The model of the FETP is “learning by doing,” through which a selected group of Ministry of Health public health professionals, called residents, participate in a combination of classroom instruction (25%) and fieldwork (75%) [3]. FETP curricula can be individualized to fit the needs of the country, but the common goal of each program is to improve surveillance systems, outbreak investigations, disease response, and data reporting. A large part of the program is the field component, which exposes the residents to real-time experiences where they learn to detect and respond to diverse public health events. On graduation from the program, the residents are skilled in applied epidemiology and are highly qualified for government-level public health positions; globally, more than 80% of FETP graduates work in government and many obtain leadership positions within national health systems [1].

The Jordan FETP (J-FETP) was established in 1998 with funding from the US Agency for International Development’s Jordan mission and with technical assistance from the CDC. The first cohort graduated 6 residents at the completion of the 2-year training. The J-FETP was supported by a CDC-assigned Resident Advisor from 1998 until 2007.

Since its inception in 1998, the J-FETP has been significantly transformed and expanded through 4 distinct phases. The initial phase, Jordan Data for Decision Making (J-DDM) Project (phase I), which ran from 1998 to 2001, comprised 2 separate programs: the J-FETP and the J-DDM program. The focus at that time was to improve the use of data at all levels of the Jordan Ministry of Health. From 2001 to 2004, the FETP was in its second phase as the Jordan Surveillance Project (phase II) and expanded its scope to include communicable and noncommunicable disease surveillance. Two major systems, the Mortality Surveillance System and the Behavioral Risk Factors Surveillance System, were put in place. In 2004 (phase III), the program was renamed the Jordan Applied Epidemiology Project, with an enhanced focus on surveillance systems. The current phase (phase IV) of the FETP is marked by the departure of the CDC-assigned Resident Advisor, demonstrating a sustainable and institutionalized J-FETP. As of 2007, the program became fully sustained and is being run by the Jordan Ministry of Health.

**Figure 1:**
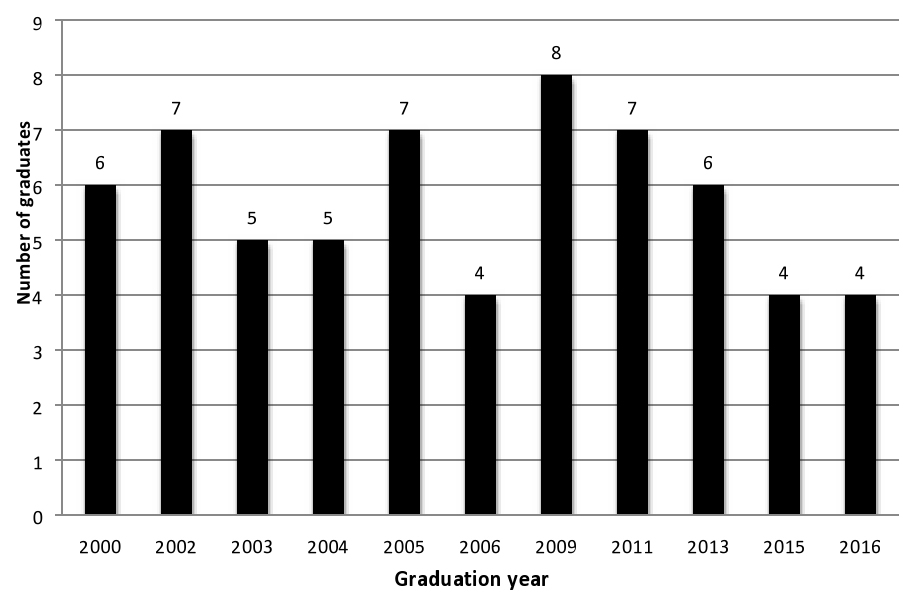
Number of graduates in the Jordan Field Epidemiology Training Program by year.

Today, the J-FETP is housed within the Ministry of Health in the Primary Health Care Administration. The program is led by the J-FETP Coordinator, a Ministry of Health official. The program uses the standard CDC FETP curriculum with modifications and case studies based on needs assessments of Jordan’s public health status. By 2017, the program had graduated a total of 63 physicians (Figure 1), with 17 residents in training. All residents who have enrolled in the program so far have been physicians, with the exception of 2 veterinarians, who enrolled during the height of the influenza epidemic in 2007. Of the graduates, 62% (39/63) work as epidemiologists at the central or governorate level of the Jordan Ministry of Health. Jordan is one of the few countries in the region that meets the international public health standard of 1 field epidemiologist per 200,000 people, with at least one FETP graduate working in 8 of the 12 governorates. All of the remaining graduates work in the region: 16% (10/63) at the government level, 13% (8/63) as regional epidemiologic experts, and 10% (6/63) with international nongovernmental organizations. The J-FETP is currently training its 13th cohort, with 14 physicians enrolled. The Ministry of Health recently incorporated the FETP into the Community Medicine Residency Program as part of the Jordan Medical Council. As a result, the J-FETP program is now accredited as a training program for the Jordanian Board Certificate in community medicine. 

 Graduates of FETP will have completed 2 years toward the community medicine certificate, creating an additional financial and career benefit for FETP residents. Integration of FETP and the Jordanian Board Certificate in community medicine has made the program an appealing option for young and motivated physicians.

Up to the end of 2017, a total 63 persons had graduated from the FETP program and 17 persons are currently enrolled in the residency program. Of the total graduates, 54 were from Jordan, 2 from Palestinian territories, 2 from Iraq, 3 from Yemen, and 2 from Syria. All were physicians except for 2, who were veterinaries. Of all graduates, 62% (39/63) are working at the central or governorate level of the national health system. All graduates who are at the Ministry of Health have management positions. A total of 6 graduates are working with international organizations.

Here we describe the J-FETP, its role in building the epidemiologic capacity of Jordan’s public health workforce, and its activities and achievements.

## The Jordan Field Epidemiology Training Program

The major function of the J-FETP is to improve reporting and surveillance systems, and prepare the country for outbreak investigations.

### Outbreak Investigations

J-FETP graduates and residents are critical contributors to outbreak investigation in Jordan and the region. J-FETP residents are trained in proper outbreak investigation practices as part of the FETP curriculum, and residents as well as graduates are called upon regularly to investigate and respond to public health issues related to crises and emergencies.

The J-FETP residents and graduates are able to quickly detect outbreaks, collect and interpret data, then communicate with the Primary Health Care Administration on the proper response to disease outbreaks. With assistance from CDC’s Outbreak Response and Prevention Branch and the World Health Organization, the J-FETP and the Ministry of Health established 5 sentinel sites in Jordan to detect and respond to foodborne illness.

The J-FETP has been a major resource in the investigation of the regional outbreak of Middle East respiratory syndrome coronavirus (MERS-CoV). In collaboration with the CDC, Jordan Ministry of Health, and the Eastern Mediterranean Public Health Network (EMPHNET), J-FETP has formed a Jordan MERS-CoV team. The J-FETP has conducted retrospective serologic and epidemiologic studies of the virus, resulting in an improved understanding of the etiology and mode of transmission of MERS-CoV, nationally and globally. J-FETP contributed to 2 investigative reports on MERS-CoV, published by the Oxford University Press. The first, “Hospital-associated outbreak of Middle East respiratory syndrome coronavirus: a serologic epidemiologic, and clinical description,” found that 9 cases of a hospital-associated respiratory illness outbreak in Jordan in 2012 were positive for MERS-CoV [4]. A second report, “Stillbirth during infection with Middle East respiratory syndrome coronavirus,” investigated the first recorded occurrence of stillbirth during infection with MERS-CoV [5]. J-FETP coleads the MERS-CoV outbreak investigation in the country.

### The Syrian Refugee Crisis

As of a July 2014, there are over 600,000 Syrian refugees in Jordan, with the majority living in host communities throughout Jordan. The Jordan Ministry of Health provides free health care to Syrian refugees in Jordan to support the health needs of the displaced population. The Ministry of Health facilities experience the burden of addressing the unique health profile of Syrian refugees and require support in optimizing their capacity. The J-FETP established a system for collecting and reporting data regarding Syrian refugee care at Ministry of Health facilities. An FETP focal point in local governorates reports data regarding refugee health care at Ministry of Health facilities to the Primary Health Care Administration. The data inform the Jordan Ministry of Health of health trends among the Syrian population such as potential disease outbreaks, access to Ministry of Health facilities by the refugee population, and the type of care provided. With this information, the Jordan Ministry of Health is able to respond to the needs of the Syrian refugee population, prevent major population health issues, and identify needs for increased health facility capacity. The Ministry of Health is also able to communicate monthly data reports to international nongovernmental organizations to enable a collaborative humanitarian response.

### Improvements in Surveillance Systems

The J-FETP has made advancements in health data surveillance that are well recognized in the Eastern Mediterranean Region. From the start of the J-FETP program in 1998, residents began to evaluate and plan for improvements in Jordan’s health surveillance systems as part of their fieldwork and training. Efforts were made to improve the collection and analysis of, and response to, surveillance data.

Mortality and morbidity data are an essential component of health information systems and are essential in identifying national and local health needs [6]. It has been estimated that noncommunicable diseases, including cardiovascular disease, cancer, diabetes, and chronic respiratory diseases, account for 60% of total deaths in Jordan [7]. J-FETP has recognized the burden of noncommunicable diseases, assessed the trend of major diseases, and addressed the concern at the national and local government levels [8]. J-FETP has led the implementation of 3 Behavioral Risk Factors Surveillance System surveys to identify the population behaviors causing noncommunicable diseases and to assess the changes in the pattern of noncommunicable diseases. The survey was designed and conducted by J-FETP residents in 2002, 2004, and 2007. Major findings of the 2002 and 2004 surveys were published in the CDC’s *Morbidity and Mortality Weekly Report* [9,10], and the 2007 survey findings were published in the CDC’s *Preventing Chronic Disease* journal [11]. The survey results were accessed by public health officers to provide government and policy decision makers with evidence-based information used in the context of determining national health priorities, as well as planning, evaluating, and monitoring country health programs. In response to the survey findings, the Non-Communicable Disease Directorate was established in the Ministry of Health in 2005. The Directorate is responsible for monitoring noncommunicable disease surveillance and implementing noncommunicable disease reduction programs. The Directorate is led by a J-FETP graduate, and J-FETP residents spend a portion of their training working within the Directorate.

Moreover, improvement in the surveillance systems resulted in appropriate data to study the trends of diseases and assess their burden. Such data have been used by many investigators who studied the disease trends and published their research in reputable journals. Recently, in 2017, 2 papers were published on the trends of cancer using the surveillance data [12,13].

The J-FETP has played a vital role in improving mortality data in Jordan [6]. J-FETP residents have examined and analyzed mortality data systems and death certificates in Jordan. The findings of the J-FETP projects led to a major review of mortality surveillance and a national effort to establish a modified system. In 2001, the Jordanian parliament passed a civil registration law that regulated death reporting, burial permits, and death certificates based on the recommendations of the J-FETP, demonstrating the feasibility of updating a national mortality statistics system. The death notification form was revised to comply with international standards, and the Jordan mortality surveillance system has been presented at a number of international conferences [8]. The updated procedures required better accuracy and completeness of reporting. Today, the Mortality Surveillance Unit is headed by a J-FETP graduate.

As a result, residents and graduates have been recognized for their applied epidemiology projects in surveillance systems in a number of international conferences, including events hosted by Training Programs in Epidemiology and Public Health Inventions Network, EMPHNET, the International Epidemiological Association, and the CDC Behavioral Risk Factors Surveillance System. Notable applied epidemiology projects include outbreak investigations, and evaluation of surveillance systems, mortality, noncommunicable diseases, and injuries.

### The Jordan Infectious Disease Information System

The J-FETP recognized the need for surveillance and reporting at the local levels, as well as strong communication to the central government. For this reason, the Jordan Infectious Disease Information System (JIDIS) was created. The electronic database was installed at the local and central directorates to track cases of infectious disease. All cases of infectious disease are recorded by Ministry of Health facility staff in the JIDIS.

Each week, the FETP residents collect, examine, and organize the data from the JIDIS into a presentation for the Directorate of Communicable Diseases at the Ministry of Health. The weekly presentation communicates all cases of infectious disease throughout the country, by governorate. During each meeting, Ministry of Health officials analyze the data and discuss action or follow-up needed on any issues of concern. The central national laboratory attends the meetings as well, in order to enhance coordination between the epidemiologists and laboratory staff. Based on the information from the JIDIS, J-FETP residents produce communicable disease reports, which are published on the Ministry of Health website.

### The Jordan Data for Decision Making Program

Established at the same time as the J-FETP, the J-DDM program was put in place to increase the effective use of data in setting health priorities and policies in Jordan. The program, which is supported by the CDC, encourages making cost-effective decisions on the allocation of resources to optimize the capacity of Jordan’s health system. While the FETP program is a 2-year program primarily for physicians who will transition into leadership positions in the government, the J-DDM program is a 12-month on-the-job training program with 5 to 6 weeks of classroom instruction, catered to midcareer health officers at the governorate and district level. These health professionals are trained in basic epidemiology, surveillance, data collection, and analysis.

The J-DDM program and J-FETP function cohesively with one another, as joint classroom sessions are conducted when appropriate. Additionally, the J-FETP residents act as facilitators and trainers throughout the J-DDM program instruction, in addition to acting as mentors to the J-DDM participants.

The J-DDM program has graduated 53 midlevel professionals through 3 cohorts since its establishment in 1998. In recent years, the J-DDM program has contributed to progressive policy changes with regard to national health. The program has trained Ministry of Health hospital personnel and established a hospital infection control surveillance system at the local levels. Notable examples of J-DDM research are a project in Balqa, Jordan, that led to increased reporting of modifiable disease from the private sector, and a report on rubella among government school teachers that resulted in revision to the national policy on teachers’ sick leave.

### Regional Contributions

The J-FETP is a major source of support in public health and epidemiology in the Eastern Mediterranean Region. The J-FETP has supported regional investigations, response, and surveillance through resident and graduate expertise, and has hosted a number of residents from neighboring FETP programs.

In 2002, the J-FETP hosted 2 residents from the Palestinian territories, 1 resident from Gaza and 1 from the West Bank. The residents completed their first year of training with the J-FETP and then returned to their homes, where they completed their second year while conducting fieldwork on surveillance. After the program, each graduate went on to lead surveillance units in Gaza and the West Bank. J-FETP supported Iraq in increasing its public health capacity through enrolling 3 Iraqi physicians into the program. The 3 residents completed the program alongside their Jordanian counterparts, during which time they conducted and published investigations in Iraq on thallium poisoning (in 2008) [14] and a cholera outbreak in 2008 (F Al-lami, written communication, December 2017). J-FETP continued to offer assistance and guidance to Iraq as it established its own FETP in 2010, led by one of the Iraqi graduates of the J-FETP. Moreover, the J-FETP hosted 3 Yemeni residents. During their stay in Jordan at the Ministry of Health, Yemeni residents in turn participated in outbreak investigations in Jordan, diversifying their skills and obtaining exposure to a range of public health concerns. The Yemeni residents completed their second year in their home country, bringing their new skills and experience back to their national Ministry of Health. The residents went on to lead a major investigation of a dengue fever outbreak and presented a comprehensive report of dengue fever along the Red and Arabian seas at the EMPHNET regional conference in 2011 [15]. In addition, J-FETP also enrolled 2 Syrians as residents in the program in 2011 with a long-term plan of starting an FETP in Syria. Though the current Syrian crisis has halted efforts to establish an FETP, Jordan remains supportive of public health professionals in neighboring countries.

Beyond training residents from countries in the region, the J-FETP has extended its support to regional outbreak investigations. The J-FETP sends graduates and residents to countries throughout the region to participate in multinational investigations in coordination with the World Health Organization and international nongovernmental organizations. EMPHNET, the network that supports the strengthening of FETPs in the Eastern Mediterranean Region, is headquartered in Amman, Jordan, and therefore the J-FETP has access to additional resources to support its capacity and impact.

## Conclusion

The J-FETP has developed immensely since its inception in 1998; today the program is regarded as a model for FETPs in the region. The success of the program is a result of many factors. First, the health system in Jordan is considered to be one of the strongest in the region. The health system is widely accessed by the population, and the Ministry of Health provides 61% of all health care [16]. The health system is comparatively well funded by the national government; with 7.72% of its gross domestic product spent on health care, Jordan has among the highest public health spending levels in the Eastern Mediterranean Region. With a relatively strong Ministry of Health, the J-FETP is well supported at the central government level and can expect attention and action in response to FETP studies and findings. Second, the J-FETP has accessed a strong pool of qualified physicians to enroll and graduate from the program. A strong system of support and technical assistance from the CDC and regional organizations such as EMPHNET has provided J-FETP with resources for growth and development.

Despite the success of the J-FETP and the accomplishments of the Jordan Ministry of Health, there remains a shortage of skilled epidemiologists in Jordan. Political unrest and humanitarian crises in the region add complexity to the public health needs in Jordan and the Eastern Mediterranean Region. The prolonged existence of these regional issues will further increase the demand for an enhanced public health response, requiring experts with field experience who are trained in outbreak investigation, surveillance, and emergency response. Additionally, as Jordan witnesses an increase in the burden of noncommunicable diseases and morbidity due to injury, J-FETP must support the Ministry of Health in addressing these health issues at the national and local levels.

As J-FETP looked to enroll a new cohort in 2017, the program intensified eligibility requirements to recruit and assess the most qualified physicians in the country. The program’s primary challenge moving forward will be to ensure the sustainability of the J-FETP through appropriate internal and external funding, and ongoing improvements to the curriculum and program function.

Although this report describes the activities and achievements of J-FETP, no evaluation study has been conducted to assess and document the performance and impact of the J-FETP. Therefore, we recommended conducting a comprehensive evaluation of the J-FETP’s impact on human resources and health services in Jordan.

In conclusion, the J-FETP has contributed significantly to improvements in surveillance systems, control of infectious diseases, outbreak investigations, and the availability of reliable morbidity and mortality data in Jordan. Moreover, the program has supported public health and epidemiology in the Eastern Mediterranean Region.

## Abbreviations

CDC: Centers for Disease Control and Prevention
EMPHNET: Eastern Mediterranean Public Health Network
FETP: Field Epidemiology Training Program
J-DDM: Jordan Data for Decision Making
J-FETP: Jordan Field Epidemiology Training Program
JIDIS: Jordan Infectious Disease Information System
MERS-CoV: Middle East respiratory syndrome coronavirus
